# A Detailed Description of Locally Available Peritoneal Dialysis in a Low-Resource Setting: A Case Report

**DOI:** 10.1155/crin/3680376

**Published:** 2025-07-31

**Authors:** Niels Jig Jansen

**Affiliations:** Department of Pediatrics, Holy Family Hospital, Berekum, Ghana

**Keywords:** acute kidney injury, case report, dialysis, low-resource setting, peritoneal dialysis

## Abstract

Acute kidney injury is a potentially fatal condition, particularly in low-resource settings, where access to renal replacement therapy is limited. This creates a problem of accessibility to healthcare for many patients living in low-resource settings. A 10-year-old boy in rural Ghana presented with anuria, edema, hypertension, and seizures, and severely elevated creatinine and urea levels were measured. Local peritoneal dialysis was started on Day 4 of admission, and the patient showed significant improvement. This report shows the effectiveness including subsequent laboratory results of locally made peritoneal dialysis and gives a detailed description on how to locally make dialysis fluids and how to perform peritoneal dialysis in a low-resource setting.

## 1. Introduction

Acute kidney injury (AKI) is a serious and possible deathly condition in critically ill patients. Due to late presentation of patients, lack of resources, and high costs for hemodialysis, it is often associated with poorer outcomes in low-income countries (LICs) compared to high-income countries (HICs) [1]. The two modalities used in dialysis are hemodialysis and peritoneal dialysis. The ISPD guidelines for peritoneal dialysis in children with AKI state that there are comparable outcomes between hemodialysis and peritoneal dialysis in this population. Therefore the decision should be based on local expertise and availability [2]. Peritoneal dialysis in AKI patients is more practiced in low-resource settings because of the lower costs and minimal requirements of infrastructure such as electricity or large volumes of clean water [3, 4]. An international internet survey comparing dialysis between HIC and LIC, in 2016, showed that LIC peritoneal dialysis is the most used dialysis modality in children with AKI (68%) [5].

In 2021, hemodialysis in Ghana costed 1100 Cedi (±130 euro) for the first round of treatment including central line insertion and 400 Cedi (±50 euro) for subsequent rounds of hemodialysis. This does not include all travel expenses to the university hospital, admission costs, and medication. In an area (Bono region, Ghana), where about 40% of the population is multidimensional poor (a combination of monetary poverty, education, and basic infrastructure services), the accessibility to hemodialysis is very poor [6]. Therefore, better accessible and affordable options are important for patients with AKI living in these settings. If the peritoneal dialysis fluids and the dialysis system are locally made from widely available equipment, peritoneal dialysis can be much cheaper and affordable than hemodialysis in this rural setting. In the course of this article, the detailed procedure for locally made peritoneal dialysis in a low-resource setting and a case will be discussed.

## 2. Case Presentation

### 2.1. Setting

Berekum is a large town in the Bono region, and most of the population relies on agriculture for their livelihood. It is located in the mid-west of Ghana, close to the border of Ivory Coast. Berekum hospital is a private not for profit hospital with 250 beds, receiving patients from multiple districts (Berekum East, Berekum West, Dormaa East, Jaman South, and Tain and Sunyani West) in the Bono region. The wards are managed by medical officers, supervised by one specialist on each ward. The hospital functions as a referral center for lower healthcare clinics in the villages. The closest university referral center is in Kumasi (a 4–5 h drive).

### 2.2. Case

A 10-year-old boy presented himself in a rural Ghanaian hospital with since 3 days increasing generalized edema, reduced urine output, and since today self-aborting seizures. The parents reported a history of nephrotic syndrome 4 years ago, but documentation was missing. There was no history of malnutrition. On examination, the blood pressure was 180/120 mmHg, there was pitting edema reaching both knees, and peri-orbital edema. The mid-upper-arm circumference was within normal limits. Urine dipstick showed positive proteins, glucose, leucocytes, blood, and urobilirubin. Creatinine and urea levels were elevated (see Table 1), malaria and HIV were negative, and liver function tests were normal, except for a low albumin. There was severe anemia with a hemoglobin level of 2.8 mmol/L. The patient was admitted with the working diagnosis of AKI to chronic kidney injury in a known patient with nephrotic syndrome with severe anemia to rule out nephritic syndrome. At the OPD, amlodipine 10 mg OD, methyldopa 250 mg BD, furosemide 40 mg TDS, prednisolone 60 mg OD, ciprofloxacin 200 mg BD, and penicillin V 250 mg BD were started before admission, and the patient was transfused with one unit of blood. The posttransfusion hemoglobin increased to 5.0 mmol/L. On the fourth day of admission, the patient experienced increasing edema and anuria, and urea and creatinine levels increased further (see Table 1). After discussion with the patient and parents, the decision was made to start peritoneal dialysis, mainly because of the inability to send the patient for referral due to financial restrains. The procedure was explained and informed consent was gained from the parents. An improvement in clinical condition, edema, urine output, and kidney function was observed during the peritoneal dialysis (Table 1). In the first 5 days of dialysis (until Day 8 admission), high ultrafiltration rates were seen. From Day 6 (Day 9 on admission), some days showed positive rates, others negative rates. The peritoneal dialysis was stopped after 11 days (Day 14 on admission) because of improving creatinine and urea levels and patients/family's request for discharge.

### 2.3. The Procedure

**Figure 1:**
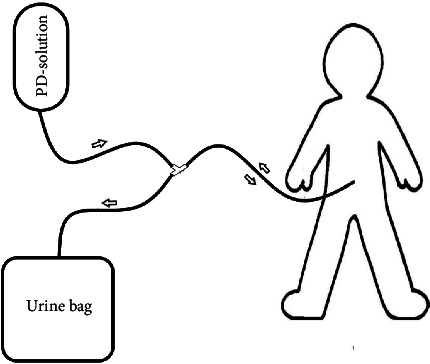
Setup of peritoneal dialysis.

We start by explaining the procedure and gaining informed consent. The procedure is performed in a procedure room on the pediatric ward, and the patient is connected to a monitor. We administer 1 g of ceftriaxone IV 30 min before the start of the procedure as prophylaxis. A urinary catheter is put in to ensure an empty bladder and monitor further urine output. The procedure was performed by a medical officer from the surgical ward and the pediatrician, a pediatric nurse was in the room for patient monitoring. We chose to give ketamine IV (1 mg/kg) as an anesthetic and put the patient on constant monitoring. A sterile field is created, and the NG tube is measured from the umbilicus to the pubic bone and marked. The NG tube is prepared as a drain by cutting additional holes for draining at the distal end. A small incision is made 1 cm infra-umbilical, and the subcutis is carefully spread till the linea alba is reached. The fascia of the linea alba is incised and the underlying peritoneum is grasped with 2 artery forceps and lifted before cutting and entering the abdominal cavity. The NG tube is inserted and aimed towards the pouch of Douglas until the marked point .We close the fascia of the linea alba tightly around the drain to minimize leakage. We close the skin with a purse-string suture and secure the NG tube with this suture. We connect the NG tube to a 3-way connector and the other ends with the dialysis solution (in) and a urine bag (out). We perform a test run of the dialysis solution for leakage. In case of leakage, we try to tunnel the NG tube (see Figure 1 for a schematic drawing of the system).

### 2.4. Solutions

A desirable dialysis solution should consist of the following; a base (lactate or bicarbonate), an osmotic agent (glucose, amino acid, or icodextrin), calcium, sodium (lower than plasma level), and water to make up the solution [2].

There are several ready-to-use solutions for peritoneal dialysis. Although most of them are not widely available in low-resource settings, Ringer's lactate on the other hand is widely available and because of its composition suitable to create a pharmaceutical-like solution (see Table 2). There are different concentrations of solutions available, mostly used are 1.5%, 2.5%, or 4.5%. The higher the concentration, the more the osmotic pressure and the stronger the pull of excess body water in a hypervolemic state. In patients with significant fluid retention, a higher concentration can be used as the fluid of choice.

To make a suitable peritoneal dialysis solution from Ringer's lactate, we need to add glucose. Taking 15 mL Ringer's lactate out of a 500 mL Ringer's lactate bottle and replacing it with 15 mL of 50% dextrose gives a comparable glucose level to the Ringer's lactate as a 1.5% pharmaceutical solution. The same can be performed with 25 mL or 45 mL to create 2.5% or 4.5%, respectively [7]. To lower the risk of peritonitis, it is common to add 125 mg/L (62.5 mg per 500 mL bottle of the 1.5% Ringer's lactate) of ceftriaxone [2, 8]. It is also a common practice to add 250 IU of heparin (500 IU/L) to each 500 mL 1.5% Ringer's lactate solution to prevent fibrin clots from blocking the catheter [2, 9].

The 1.5% Ringer's lactate solution will not be very effective in lowering the potassium levels, but it will also not make them rise too much. If due to the fluid retention, there is a serum potassium of < 4.0 mmol/L, the addition of KCl to the solution can be considered [2].

### 2.5. Dialysis

The amount of solution used is weight-dependent (10 mL/kg for children and 20–30 mL/kg for adults). Every hour, the 1.5% Ringer's lactate solution is quickly inserted into the abdomen, and the 3-way connector is closed. After 1 h, the fluid is drained from the abdomen into the urine bag, and the amount of ultrafiltered fluid is measured and noted. After everything is drained, a new dialysis solution is quickly inserted into the abdomen for 1 h. This is repeated every hour. Every 12 or 24 h, the total ultrafiltered fluids and urine production are calculated and noted. Dialysis should be continued until a significant improvement in creatinine levels is seen or if the urinary output improves to > 1.5 mL/kg/h.

## 3. Discussion

Studies on peritoneal dialysis in low-resource settings and the preparation of dialysis solutions are available. However, none of these studies gives a detailed description on how to apply peritoneal dialysis in a low-resource setting. The case clearly shows the benefits of peritoneal dialysis with a decrease in creatinine and an increase in urine output. In case of a pure AKI, only 3–5 days of peritoneal dialysis would be needed before the kidneys are able to recover and start working again.

Although the procedure is not a difficult one, there are a few points to take under consideration. First of all, it is very time-consuming and labor-intensive for the ward staff, which makes it very important to involve them in the management. Second, there is a risk of fluid leakage through the insertion. If the leakage is severe, it is uncomfortable for the patient and makes the ultrafiltration calculation unreliable. The urine output and creatinine levels are still the main parameters, and they are not influenced by the leakage. To reduce the leakage, it is advisable to tunnel the peritoneal dialysis tube. Third, it is advisable to monitor for hyperglycemia, especially when higher concentrations of Ringer's lactate–dextrose solutions are used. Finally, care should be taken in case of liver failure. Ketamine, used during the insertion of the tube, is broken down by the liver. In case of liver failure, ketamine can cause more damage, and therefore other anesthetics are advised. Furthermore, the lactate in commercial peritoneal dialysis solution or Ringer's lactate should be avoided in liver failure. In this case, a bicarbonate solution is preferred. A basic bicarbonate 2.5% solution can be made by mixing 500 mL of dextrose 5%, 500 mL of normal saline 0.9%, 40 mL of NaHCo_3_ 8.4%, 7.5 mL of sodium chloride 20%, and 8 mL of calcium gluconate 10%. A more detailed description of the bicarbonate and other dialysis solutions is outside the scope of this article.

Unfortunately, documentation and follow-up remain a challenge in low-resource settings such as ours. Therefore the data on the following topics are less detailed than we wished; the history of previous admission 4 years ago, the duration of onset of symptoms this time, urine output monitoring before dialysis, and follow-up after stopping peritoneal dialysis. Also, the rationale for prescribing ciprofloxacin and penicillin at the OPD remains unclear. This should be seen in the scope of empirical antibiotic misuse, which is common in low-resource settings.

## 4. Conclusion

This report shows the effectiveness of locally made peritoneal dialysis and gives a detailed description on how to locally make dialysis fluids and how to perform peritoneal dialysis in a low-resource setting. Especially in the primary and secondary facilities of low-resource settings, a significant volume of patients is seen, and these facilities are commonly staffed by doctors working across multiple disciplines without specialist training. This detailed description and the possible pitfalls described in this article can help with the availability and accessibility of peritoneal dialysis for patients with AKI.

## Figures and Tables

**Table 1:** Dialysis and laboratory results.

	Ultrafiltration dialysis	Urine output	Creatinine (umol/L) [59–104]	Urea (mmol/L) [1.8–6.4]	Sodium (mmol/L) [135–155]	Potassium (mmol/L) [3.5–5.5]	Chloride (mmol/L) [98–108]
Day 1 (admission)			465.4	16.3			
Day 4 (12 h) (PD start)	+3300 mL (+11 mL/kg/h)	20 mL (0.03 mL/kg/h)	1116.45	20.5	137	3.0	110
Day 5	+3200 mL (+5.8 mL/kg/h)	110 mL (0.18 mL/kg/h)					
Day 6	+1955 mL (+3.55 mL/kg/h)	70 mL (0.12 mL/kg/h)					
Day 7	+1145 mL (+2.1 mL/kg/h)	^∗^					
Day 8	+280 mL (+0.44 mL/kg/h)	^∗^	711.9	17.9	122	4.9	96
Day 9	−330 mL (−0.55 mL/kg/h)^∗∗^	380 mL (0.63 mL/kg/h)					
Day 10	−1460 mL (−2.78 mL/kg/h)^∗∗^	400 mL (0.67 mL/kg/h)					
Day 11	−230 mL (−0.4 mL/kg/h)	600 mL (1.0 mL/kg/h)					
Day 12	+1980 mLs (+3.3 mL/kg/h)	400 mL (0.67 mL/kg/h)					
Day 13	+170 mLs (+0.28 mL/kg/h)	300 mL (0.5 mL/kg/h)					
Day 14	−880 mLs (−1.47 mL/kg/h)	400 mL (0.67 mL/kg/h)	507.0	14.6	121	4.6	93

^∗^No reliable urine output monitoring due to a leaking urine catheter.

^∗∗^Severe leaking from the drainage site, therefore underestimation of the total ultrafiltration.

**Table 2:** Composition of 1.5% commercial peritoneal dialysis solution and Ringer's lactate.

	1.5% Commercial peritoneal dialysis solution	Ringer's lactate
Na^+^	132 mmol/L	130 mmol/L
K^+^	Nil	4 mmol/L
Cl^−^	96.0 mmol/L	109 mmol/L
Ca^2+^	2.5 mmol/L	2.7 mmol/L
Mg^2+^	0.5 mmol/L	Nil
Lactate	40 mmol/L	28 mmol/L
Glucose	15 g/L	Nil^∗^

^∗^30 mL dextrose 50% = 15 g glucose.

## Data Availability

The data that support the findings of this study are available on request from the corresponding author. The data are not publicly available due to privacy or ethical restrictions.

## References

[1] Olowu W. A., Niang A., Osafo C. (2016). Outcomes of Acute Kidney Injury in Children and Adults in Sub-Saharan Africa: A Systematic Review. *Lancet Global Health*.

[2] Nourse P., Cullis B., Finkelstein F. (2021). ISPD Guidelines for Peritoneal Dialysis in Acute Kidney Injury: 2020 Update (Paediatrics). *Peritoneal Dialysis International: Journal of the International Society for Peritoneal Dialysis*.

[3] Cullis B. (2023). Peritoneal Dialysis for Acute Kidney Injury: Back on the Front-Line. *Clinical Kidney Journal*.

[4] Niang A., Iyengar A., Luyckx V. A. (2018). Hemodialysis Versus Peritoneal Dialysis in Resource-Limited Settings. *Current Opinion in Nephrology and Hypertension*.

[5] Raina R., Chauvin A. M., Bunchman T. (2017). Treatment of AKI in Developing and Developed Countries: An International Survey of Pediatric Dialysis Modalities. *PLoS One*.

[6] Ghana Statistical Service (2022). Annual Household Income and Expenditure Survey (AHIES). https://statsghana.gov.gh/gssmain/fileUpload/pressrelease/AHIES%2520executive%2520summary%25201%2520(3_24PM).pdf.

[7] Palmer D., Lawton W. J., Barrier C. (2018). Peritoneal Dialysis for AKI in Cameroon: Commercial Vs Locally-Made Solutions. *Peritoneal Dialysis International: Journal of the International Society for Peritoneal Dialysis*.

[8] Warady B. A., Bakkaloglu S., Newland J. (2012). Consensus Guidelines for the Prevention and Treatment of Catheter-Related Infections and Peritonitis in Pediatric Patients Receiving Peritoneal Dialysis: 2012 Update. *Peritoneal Dialysis International: Journal of the International Society for Peritoneal Dialysis*.

[9] Bonilla-Félix M. (2013). Peritoneal Dialysis in the Pediatric Intensive Care Unit Setting: Techniques, Quantitations and Outcomes. *Blood Purification*.

